# Machine Learning Model for Predicting Multidrug Resistance in Clinical *Escherichia coli* Isolates: A Retrospective General Surgery Study

**DOI:** 10.3390/antibiotics14100969

**Published:** 2025-09-26

**Authors:** Hüseyin Kerem Tolan, İrfan Aydın, Handan Tanyildizi-Kokkulunk, Mehmet Karakuş, Yüksel Akkaya, Osman Kaya, Ferruh Kemal İşman

**Affiliations:** 1 Department of General Surgery, Ümraniye Training and Research Hospital, University of Health Sciences, 34668 Istanbul, Türkiye; 2 Pharmacy Services Program, Pharmacy Services Department, Health Services Vocational School, Fenerbahçe University, 34758 Istanbul, Türkiye; irfan.aydin@fbu.edu.tr; 3 Department of Arts & Sciences, Maine Maritime Academy, Castine, ME 04420, USA; handan.kokkulunk@mma.edu; 4 Department of Medical Microbiology, Hamidiye Faculty of Medicine, University of Health Sciences, 34668 Istanbul, Türkiye; mehmet.karakus@sbu.edu.tr (M.K.); yuksel.akkaya@sbu.edu.tr (Y.A.); 5 Department of Biochemistry, Göztepe Prof. Dr. Süleyman Yalçın Training and Research Hospital, 34730 Istanbul, Türkiye; osman.kaya@medeniyet.edu.tr (O.K.); ferruh.isman@medeniyet.edu.tr (F.K.İ.)

**Keywords:** antibiotic resistance, *Escherichia coli*, machine learning, random forest

## Abstract

**Background/Objectives**: *Escherichia coli* is one of the leading causes of surgical site infections (SSIs) and poses a growing public health concern due to its increasing antimicrobial resistance. High rates of extended-spectrum beta-lactamase (ESBL) production among *E. coli* strains complicate treatment outcomes and emphasize the need for effective surveillance and control strategies. **Methods**: A total of 691 *E. coli* isolates from general surgery clinics (2020–2025) were identified using MALDI-TOF MS. Antibiotic susceptibility data and patient variables were cleaned, encoded, and used to predict resistance using the Random Forest, CatBoost, and Naive Bayes algorithms. SMOTE addressed class imbalance, and model performance was assessed through various validation methods. **Results**: Among the three machine learning models tested, Random Forest (RF) showed the best performance in predicting antibiotic resistance of *E. coli*, achieving median accuracy, precision, recall, and F1-scores of 0.90 and AUC values up to 0.99 for key antibiotics. CatBoost performed similarly but was less stable with imbalanced data, while Naive Bayes showed lower accuracy. Feature importance analysis highlighted strong inter-antibiotic resistance links, especially among β-lactams, and some influence of demographic factors. **Conclusions**: This study highlights the potential of simple, high-performing models using structured clinical data to predict antimicrobial resistance, especially in resource-limited clinical settings. By incorporating machine learning into antimicrobial resistance (AMR) surveillance systems, our goal is to support the advancement of rapid diagnostics and targeted antimicrobial stewardship approaches, which are essential in addressing the growing challenge of multidrug resistance.

## 1. Introduction

*Escherichia coli* (*E. coli*) is a facultative anaerobic, Gram-negative bacterium belonging to the Enterobacteriaceae family; it constitutes a significant part of the normal intestinal flora both in humans and animals [1]. While most *E. coli* strains are harmless commensals, certain pathogenic variants can cause a wide spectrum of clinical conditions, including urinary tract infections (UTIs), neonatal meningitis, sepsis, and various intestinal diseases [2]. *E. coli* is also a major cause of nosocomial infections, particularly catheter-associated UTIs and bloodstream infections [3].

The rising prevalence of antibiotic resistance in *E. coli* represents a global public health challenge. Extended-spectrum beta-lactamase (ESBL)-producing and carbapenem-resistant *E. coli* strains are increasingly reported worldwide, limiting treatment options and contributing to higher morbidity and mortality [4,5,6]. According to recent epidemiological data, the prevalence of ESBL-producing *E. coli* has reached alarming levels in some regions: 58% in India, 64% in Egypt, and 79% in Türkiye [7,8,9]. More specifically, surveillance studies from general surgery wards in Türkiye have reported ESBL production in up to 91.3% of *E. coli* isolates, raising serious concerns about treatment outcomes. Such resistance trends result in delays in initiating effective therapy, longer hospital stays, and increased healthcare costs [10].

Conventional antimicrobial susceptibility testing (AST), including broth microdilution and disk diffusion, requires 24–72 h to provide results, which limits its value in urgent clinical decision making. Inappropriate empirical antibiotic use during this waiting period often contributes to poor patient outcomes and the acceleration of resistance selection [11]. Therefore, rapid alternative strategies for predicting resistance patterns are urgently needed.

Recent advances in machine learning (ML) have shown promise in addressing these challenges by enabling rapid, data-driven predictions of antimicrobial resistance (AMR). ML models trained on genomic, phenotypic, or mass spectrometry data have demonstrated high accuracy in identifying resistance mechanisms and predicting susceptibility profiles across several bacterial species, including *E. coli* [3,11,12,13]. For example, algorithms such as Support Vector Classifier (SVC), Random Forest (RF), Decision Tree (DT), and K-Nearest Neighbors (KNN) have been successfully applied to classify resistant *E. coli* isolates and infer resistance determinants from whole-genome sequencing or MALDI-TOF MS spectra. These models not only enhance prediction performance but also offer interpretable outputs that can be valuable in clinical decision making [14,15,16].

In general surgery departments, where patients frequently undergo invasive procedures and prolonged hospitalization, *E. coli* remains a leading cause of healthcare-associated infections, particularly surgical site infections (SSIs), UTIs, and bacteremia. The increasing prevalence of multidrug-resistant *E. coli* strains including ESBL and carbapenemase producers has made empirical antibiotic selection increasingly uncertain, frequently resulting in inappropriate initial therapy and worse outcomes. Rapid and accurate identification of resistance patterns is therefore crucial for optimizing perioperative prophylaxis and guiding early therapeutic interventions. In this context, the integration of ML models trained on phenotypic and mass spectrometry data offers a powerful alternative for the early prediction of resistance profiles in *E. coli* isolates.

The present study aims to utilize ML algorithms to classify *E. coli* isolates from general surgery wards based on their antibiotic susceptibility profiles, thereby facilitating early detection of resistance and supporting personalized therapy choices. By integrating ML into AMR surveillance frameworks, we seek to contribute to the development of rapid diagnostic tools and precision antimicrobial stewardship strategies, which are essential in the era of rising multidrug resistance. This study addresses an understudied setting by providing locally relevant predictive models. To best of our knowledge, this is the first ML study in Türkiye to employ multi-hospital general surgery data for resistance prediction in *E. coli*. We further contend that the methods and data presented here will support the advancement of hospital information systems and facilitate the adaptation of ML approaches across diverse healthcare institutions.

## 2. Results

**Figure 1:**
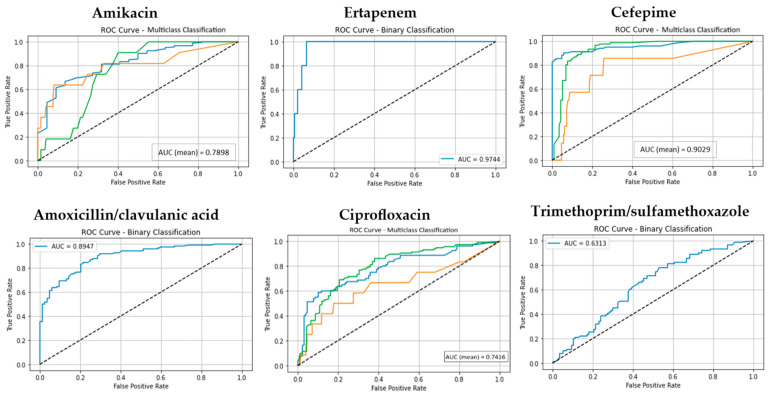
The ROC curves and corresponding AUC values for the Random Forest (RF) model across for six antibiotic groups. (Classes 1, 2, and 3 are shown in blue, orange, and green, respectively. The diagonal dotted lines represent the performance of a random classifier (AUC = 0.5), serving as a baseline for comparison).

**Figure 2:**
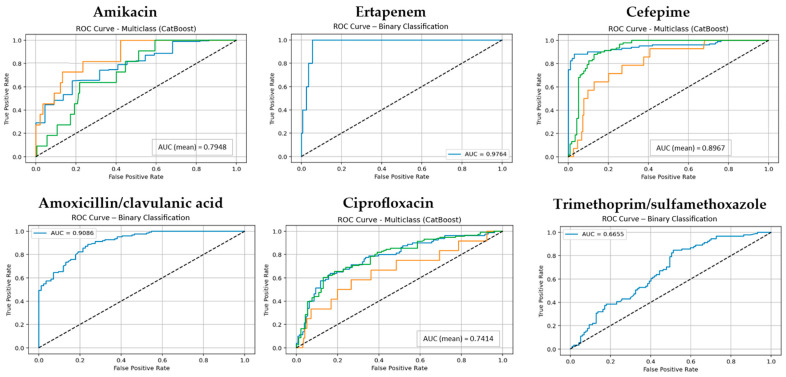
The ROC curves and corresponding AUC values for the CatBoost model across for six antibiotic groups. (Classes 1, 2, and 3 are shown in blue, orange, and green, respectively. The diagonal dotted lines represent the performance of a random classifier (AUC = 0.5), serving as a baseline for comparison).

**Figure 3:**
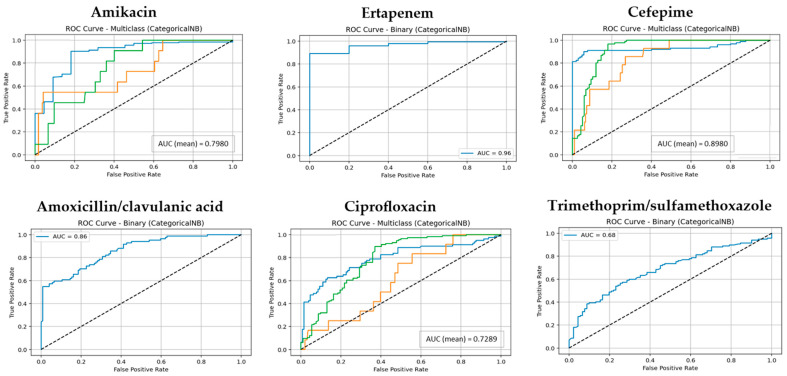
The ROC curves and corresponding AUC values for the NB model across six antibiotic groups. (Classes 1, 2, and 3 are shown in blue, orange, and green, respectively. The diagonal dotted lines represent the performance of a random classifier (AUC = 0.5), serving as a baseline for comparison).

The predictive performance of three supervised machine learning algorithms, RF, Boosted Logistic Regression (CatBoost), and Naive Bayes (NB), was evaluated in estimating the antibiotic resistance profiles of *E. coli* isolates. Performance was assessed across three validation scenarios: (i) a 70:30 train–test split, (ii) 5-fold cross-validation, and (iii) 10-fold cross-validation. Each antibiotic was modeled as a separate classification task, and model evaluation was conducted using key metrics including accuracy, precision, recall, F1-score, and area under the ROC curve (AUC), with particular emphasis on results from 10-fold cross-validation due to its enhanced reliability. The classification performance of the RF model across all antibiotics is shown in Supplementary File S1. Additionally, for each model, the ROC curves and corresponding mean AUC values are presented in Figure 1, Figure 2 and Figure 3 using one representative antibiotic from each group (Aminoglycosides, Carbapenems, Cephalosporins, Beta-lactam/β-lactamase inhibitor combinations, Fluoroquinolones, and Sulfonamides/Trimethoprim), while the complete set of antibiotics included in the study is provided in the Supplementary File S1.

The model consistently demonstrated high predictive accuracy, particularly for antibiotics with well-balanced class distributions. In the 10-fold cross-validation scenario, Random Forest (RF) achieved median accuracy, precision, recall, and F1-score values around 0.95, underscoring its robustness in handling categorical datasets. Notably, RF achieved near-perfect AUC values (0.99) for both Ampicillin and Ertapenem, indicating excellent discrimination ability. Performance was also consistently strong for Ceftriaxone and Cefuroxime (accuracy: 0.95).

Like RF, CatBoost yielded strong results under the 10-fold cross-validation setting, with median values around 0.93. For some antibiotics such as Ceftriaxone, Ertapenem, and Ceftazidime, CatBoost matched RF and occasionally achieved marginally higher performance in individual metrics. However, for antibiotics with greater class imbalance, such as Cefoxitin and Trimethoprim/sulfamethoxazole, CatBoost exhibited less stability (accuracy around 0.68–0.70), reflecting sensitivity to distributional challenges (Supplementary File S1).

By contrast, Naive Bayes (NB) lagged both RF and CatBoost across all validation settings. Although NB achieved acceptable AUC values for certain antibiotics (e.g., 0.95 for Ceftriaxone and 0.92 for Ampicillin), its overall median metrics in the 10-fold cross-validation setting (accuracy, precision, recall, F1-score: ~0.84) were notably lower. NB also demonstrated pronounced variability across antibiotics, particularly struggling with Amikacin, Ciprofloxacin, and Trimethoprim/sulfamethoxazole, suggesting that its assumptions of feature independence limit its effectiveness in complex datasets with correlated predictors.

Furthermore, we evaluated the calibration of the Random Forest model using Brier scores to assess the agreement between predicted probabilities and observed resistance. The Brier analyses revealed meaningful variation across antibiotics. Ertapenem (Brier = 0.01), ampicillin (Brier = 0.04), ceftazidime (Brier = 0.04), cefepime (Brier = 0.05), and amikacin (Brier = 0.05) exhibited the lowest overall scores, consistent with their high AUCs, indicating that predicted probabilities were both highly discriminative and well calibrated. These findings suggest that RF-derived probabilities for carbapenems, aminoglycosides, and broad-spectrum β-lactams are clinically reliable, which is especially important where inappropriate treatment carries significant risk.

By contrast, amoxicillin/clavulanic acid (Brier = 0.13) and trimethoprim/sulfamethoxazole (Brier = 0.20) demonstrated higher scores, underscoring that high AUCs may coincide with more modest calibration. In these cases, the model effectively separates resistant from susceptible isolates but may under- or over-estimate absolute resistance probabilities.

Importantly, integrating calibration metrics alongside ROC–AUC provides a more comprehensive performance evaluation. High AUCs alone could foster overconfidence in model predictions, whereas calibration analyses reveal where probabilities may deviate from observed outcomes. For antibiotics such as ertapenem, cefepime, ampicillin, ceftazidime, and amikacin, where both discrimination and calibration were strong, the model outputs are highly actionable. For amoxicillin/clavulanic acid and trimethoprim/sulfamethoxazole, however, probability calibration may require further refinement to ensure clinical reliability.

In addition to the ensemble learning algorithms (RF and CatBoost), we evaluated a simpler statistical baseline, Logistic Regression, across all antibiotics. Logistic Regression showed considerable variability, with performance metrics strongly influenced by class imbalance and prevalence. While some antibiotics, such as Cefuroxime and Ampicillin, achieved relatively high predictive performance, others, including Ertapenem and Meropenem, performed poorly due to the very low number of resistant isolates. When aggregated across all antibiotics, Logistic Regression yielded a mean accuracy of 0.86, notably lower than Random Forest and CatBoost, underscoring the added value of advanced machine learning models in handling class imbalance and capturing complex, nonlinear relationships. These results confirm that the superior performance of RF and CatBoost reflects a substantive improvement over traditional statistical approaches.

When comparing the three models across all performance metrics and antibiotics, Random Forest consistently yielded the highest median scores and most stable performance across validation settings. It achieved the best trade-off between accuracy and AUC values, demonstrating strong predictive power and better handling of class imbalance compared to the other models, although performance for highly imbalanced antibiotics such as Cefoxitin and Trimethoprim/sulfamethoxazole was still limited. 

In addition to the conventional performance metrics (accuracy, precision, recall, F1-score, and AUC), we also calculated imbalance-sensitive measures, namely Balanced Accuracy, Cohen’s Kappa, and Matthews Correlation Coefficient (MCC), based on the 10-fold cross-validation confusion matrices of the Random Forest model. These metrics confirmed the robustness of our results across antibiotics with varying class distributions. For example, Ertapenem and Amikacin achieved particularly strong outcomes (Balanced Accuracy: 0.989 and 0.975; Kappa: 0.979 and 0.963; MCC: 0.979 and 0.963, respectively), highlighting the excellent discriminative capacity of the model even under highly imbalanced settings. Similarly, Ceftriaxone (Balanced Accuracy: 0.951, Kappa: 0.901, MCC: 0.901) and Ampicillin (Balanced Accuracy: 0.957, Kappa: 0.914, MCC: 0.914) also demonstrated consistently high agreement between predictions and true labels. On the other hand, antibiotics with more skewed distributions, such as Trimethoprim/Sulfamethoxazole (Balanced Accuracy: 0.697, Kappa: 0.395, MCC: 0.395), showed lower agreement, underscoring the challenge of extreme imbalance despite the use of SMOTE. 

To address the issue of low-prevalence outcomes, certain antibiotics, such as Meropenem (with only four resistant cases in our dataset), were affected by extreme class imbalance. Although these antibiotics were included for completeness, the corresponding results should be interpreted with caution, as the limited number of resistant isolates constrains both reliability and generalizability of the predictive performance. Consistent with best practices in antimicrobial resistance modeling, these low-prevalence antibiotics are therefore considered exploratory analyses rather than conclusive findings. In contrast, antibiotics with more balanced distributions (e.g., Ceftriaxone, Ertapenem, Ampicillin) demonstrated robust and reliable performance across multiple evaluation metrics.

Importantly, the results for intermediate cases such as Amoxicillin/Clavulanic acid (Balanced Accuracy: 0.808, Kappa: 0.617, MCC: 0.617) indicate that the model retains acceptable reliability even when the resistant class is underrepresented. Overall, the inclusion of imbalance-sensitive metrics validates that the predictive performance of the Random Forest model remains robust and clinically meaningful, while also highlighting antibiotics where future work may benefit from alternative strategies (e.g., additional data collection or multi-label approaches) to further mitigate the effects of imbalance. 

Cat-Boost closely followed RF in overall performance, and for certain antibiotics such as Ertapenem, Ceftriaxone, and Ceftazidime, it matched or even marginally outperformed RF in individual metrics. By contrast, Naive Bayes (NB), though computationally efficient, exhibited markedly lower and less consistent performance, particularly for antibiotics with skewed class distributions (e.g., Amikacin, Ciprofloxacin, TMP-SMX). Therefore, based on the observed results, RF emerged as the most optimal and generalizable model for predicting *E. coli* antibiotic resistance in this dataset. Given its superior and consistent performance across evaluation metrics, permutation-based feature importance analysis was subsequently conducted using the RF model, and additionally the CatBoost model as followed as shown in Supplementary File S1.

In this study, each antibiotic was modeled separately as a binary classification task. This decision was primarily motivated by two considerations. First, from a clinical perspective, resistance to individual antibiotics is often reported and interpreted independently in routine antibiograms, and therapeutic decisions are typically made based on the susceptibility of a specific drug. Therefore, modeling each antibiotic separately allowed us to directly align the machine learning outputs with existing clinical reporting standards and physician decision-making processes. Second, our dataset contained substantial variability in class balance across antibiotics (e.g., highly skewed distributions for cefoxitin or trimethoprim/sulfamethoxazole), which could have introduced instability if modeled jointly. Independent binary classification minimized this risk and enabled tailored handling of imbalance correction (SMOTE) and feature weighting for each antibiotic, thereby improving interpretability and robustness of predictions in a clinical setting.

Nevertheless, we acknowledge that antibiotic resistance phenotypes are not independent. Our feature importance analysis already highlighted strong inter-antibiotic associations, particularly among β-lactams, suggesting that correlated resistance profiles exist within the dataset. Multi-label learning approaches, such as classifier chains or multi-task deep learning models, could therefore capture these dependencies more explicitly by modeling resistance outcomes simultaneously. Such methods may improve predictive performance by leveraging shared resistance mechanisms and co-occurrence patterns. For example, a multi-task neural architecture could jointly optimize predictions across antibiotics, while classifier chains could sequentially propagate predicted resistances to inform subsequent outputs. Although beyond the scope of the present work, future research should explore multi-label strategies in this context, particularly as larger multicenter datasets become available. This may enable the development of models that better reflect the multidrug-resistant reality of clinical *E. coli* isolates, while still maintaining compatibility with antibiotic-specific reporting required in clinical microbiology.

In the group-based overview, permutation importance results demonstrate that the predictive contribution of features varies notably across antibiotic groups. In general, inter-antibiotic resistance patterns particularly among β-lactams (cephalosporins and β-Lactams/β-Lactamase Inhibitors) had substantial influence in predicting resistance to individual antibiotics. Cephalosporins (e.g., Ceftriaxone, Ceftazidime, Cefuroxime) were strongly interconnected, frequently emerging as top predictors for one another, which suggests correlated resistance profiles, likely due to shared mechanisms of action.

In the aminoglycoside and β-Lactams/β-Lactamase Inhibitors, demographic features such as year 2020 and sex emerged as influential, potentially reflecting temporal patterns or patient-level heterogeneity in resistance. Moreover, certain β-Lactams/β-Lactamase Inhibitors and carbapenems showed substantial cross-predictive utility, such as Piperacillin/tazobactam being a high-impact feature in multiple models (e.g., Ertapenem, Amoxicillin/clavulanic acid).

The antibiotic-specific permutation importance analysis revealed key patterns in resistance prediction. For several β-lactams particularly cephalosporins, such as Ceftriaxone, Ceftazidime, and Cefepime, mutual resistance signals were dominant, with cross-predictive importance scores exceeding +0.20, underscoring strong class-level associations. β-Lactams/β-Lactamase Inhibitors derivates such as Ampicillin and Amoxicillin/clavulanic acid also displayed high interdependence, while Piperacillin/tazobactam was notably predictive across multiple models. Temporal variables (e.g., year 2020) emerged as influential for Amikacin, Cefuroxime, and Piperacillin/tazobactam, pointing to time-sensitive shifts in resistance. In contrast, fluoroquinolone (Ciprofloxacin) and folate inhibitor (Trimethoprim/sulfamethoxazole) resistance was more influenced by demographic factors like age and sex, suggesting population-level variation. These results highlight both temporal and multidrug resistance dynamics across distinct antibiotic classes. Additionally, the table of the effect of features on classification using permutation importance in the RF model determined as the optimal algorithm is given in the Supplementary File S1.

## 3. Discussion

The increasing prevalence of multidrug-resistant *E. coli* in surgical wards poses a serious challenge to effective antimicrobial therapy, particularly in time-sensitive clinical settings such as general surgery. Our study demonstrates the feasibility and clinical relevance of applying machine learning models to predict antimicrobial resistance patterns in *E. coli*-based data. Unlike conventional culture-based methods, which are time-consuming and often delay appropriate treatment, our approach offers a rapid, scalable, and interpretable alternative that may significantly reduce diagnostic turnaround times. This is particularly valuable in general surgery departments, where early initiation of targeted therapy is critical to preventing postoperative infections and minimizing morbidity. Moreover, integrating explainable ML techniques enhances the interpretability and trustworthiness of model outputs, thereby supporting real-time clinical decision-making and strengthening hospital-level antimicrobial stewardship programs.

According to the recent reviews and research studies, *E. coli* is one of the most common healthcare associated pathogens worldwide [17]. In diverse geographic and procedural settings, *E. coli* remains a leading Gram-negative pathogen in SSIs, constituting between 12% and 48% of bacterial isolates ranging from 42.3% in gastrointestinal surgery patients to 24.5% in mixed surgical wound cohorts, and up to 47.8% in general surgical units [17,18,19,20,21,22]. Studying *E. coli* in the context of surgical infections is therefore essential as it supports the development of rapid, data-driven predictive models for antimicrobial resistance, informs empirical therapy choices, and ultimately has the potential to reduce postoperative morbidity, mortality, and healthcare costs on a global scale.

Turkish surveillance data affirm the urgency of this issue. In Türkiye, *E. coli* remains one of the most frequently isolated pathogens in SSIs, particularly following abdominal and colorectal procedures. Between 2014 and 2019, rates of ESBL production and carbapenem resistance among *E. coli* isolates in intensive care units (ICUs) increased markedly and were closely associated with higher incidences of secondary bloodstream infections [23]. A study conducted at Gaziantep University Hospital involving 1397 patients reported a postoperative SSI rate of 9.4%, with *E. coli* as the most common isolated microorganism, accounting for 32.8% of all cases [24]. Moreover, 86.3% of *E. coli* strains in this study produced ESBL, complicating treatment and elevating the risks of morbidity and mortality. Given the surgical burden of *E. coli*-related infections and the high prevalence of multidrug resistance, efforts to model and predict resistance patterns in Turkish hospitals are both timely and necessary.

In this study, we developed and validated machine learning models to predict antimicrobial resistance in *E. coli* using datasets comprising patient demographics, clinical variables, and antibiogram results from general surgery clinics across 11 hospitals in Istanbul. Our ML model is trained on five years of MALDI-TOF data, demostrating rapid and interpretable prediction of resistance, offering a practical alternative to time-consuming culture-based testing and supporting early clinical decision making. Furthermore, our findings align with the WHO-supported Tricycle project, which reported high ESBL carriage rates 49% in bloodstream isolates as a significant One Health concern [6]. Rapid ML-driven tools, particularly when explainable, can assist clinicians in making informed treatment decisions before susceptibility results become available. Among the evaluated algorithms (RF, CatBoost, and NB), the RF model achieved the best diagnostic performance with an AUROC of 0.87. Compared to recent studies in the literature, this result positions our model among the higher-performing approaches that rely on structured clinical data rather than genomic sequencing or spectral analysis (Table 1) [10].

The inclusion of Logistic Regression as a baseline further highlights the added value of ensemble machine learning methods. While Logistic Regression performed reasonably for some antibiotics, it struggled with low-prevalence outcomes and overall yielded lower accuracy than RF and CatBoost. This comparison underscores that the advanced models not only handle class imbalance more effectively but also capture complex, nonlinear relationships that simpler statistical methods cannot, reinforcing the rationale for using ML approaches in predicting *E. coli* antibiotic resistance.

Beyond discrimination metrics, we assessed the calibration of predicted probabilities using Brier scores. The calibration analyses indicated that, for antibiotics such as ertapenem, ampicillin, ceftazidime, cefepime, and amikacin, the predicted probabilities closely matched the observed resistance rates, demonstrating reliable probabilistic outputs suitable for clinical decision-making. By contrast, higher Brier scores for amoxicillin/clavulanic acid and trimethoprim/sulfamethoxazole suggested potential over- or under-estimation of absolute resistance probabilities despite good discrimination, highlighting areas where further refinement or additional data could improve clinical reliability.

The implementation of ML prediction systems has the potential to benefit multiple stakeholders of healthcare ecosystem. For patients, timely and targeted antimicrobial therapy can reduce the risk of treatment failure, postoperative complications, and prolonged hospitalizations. For clinicians, particularly surgeons and infectious disease specialists, such tools can provide real-time guidance in selecting the most appropriate empirical therapy, thereby improving decision-making in time-sensitive situations. For example, in a patient presenting with a SSI, the model could provide an early prediction of likely antimicrobial resistance in the causative *E. coli* isolate before conventional AST results are available, thereby guiding clinicians toward more appropriate empiric therapy and reducing delays in effective treatment. At the institutional and national levels, integration of predictive models into hospital information systems and surveillance networks can enhance antimicrobial stewardship, optimize resource allocation, and reduce the overall economic burden associated with multidrug-resistant infections. Ultimately, this approach supports both individual patient care and public health by contributing to the containment of antimicrobial resistance.

Our findings corroborate and extend prior work, such as that conducted by Tzelves et al. [28] who reported robust predictive accuracy using demographic and laboratory variables in ICU and emergency department cohorts. Notably, whereas previous studies have focused broadly on multidrug resistance phenotypes across heterogeneous bacterial populations or urinary tract pathogens, our approach specifically targets *E. coli*, thereby providing a species-specific, clinically relevant predictive model that can support tailored antimicrobial stewardship interventions. Moreover, the simultaneous evaluation of heterogeneous ML classifiers, including gradient boosting (CatBoost) probabilistic models (NB) and RF enables a direct comparison of algorithmic efficacy within a controlled, single-pathogen context, a methodological gap in existing antibiotic resistance prediction literature.

Compared with genomics-based predictive models utilizing whole-genome sequencing (WGS) data [3] or mass spectrometry–based approaches leveraging MALDI-TOF spectral profiles [14,16], our ML model relies on readily available clinical parameters, circumventing the need for costly sequencing infrastructure and advanced analytical platforms. Although WGS and MALDI-TOF-enabled models often achieve AUROCs exceeding 0.90 due to their high molecular resolution, our data-driven clinical model offers an accessible, scalable, and cost-efficient alternative that is more amenable to implementation in resource constrained healthcare settings.

In U.S.-based studies [25,30,31], which similarly utilize structured clinical variables, AUROC values ranging from 0.66 and 0.81 were reported, underscoring the comparatively higher discriminative capability of our model. Additionally, broad-cohort analyses by Corbin et al. [27] and Rich et al. [26] yielded more modest AUROCs (0.57–0.74), likely reflecting population heterogeneity and dataset scale. By contrast, the ML model developed by Tran Quoc et al. [11] using electronic medical records and antibiogram data achieved remarkably high predictive accuracy (AUROC 0.89–1.00) in ICU patients, highlighting the influence of ICU-specific epidemiological factors and data granularity. Importantly, the strong performance of our model using similar clinical data further supports its versatility and potential for broad clinical application.

Despite the promising results, several limitations must be acknowledged. Unlike models trained on large-scale datasets from multiple international centers [27], our dataset is limited to a single-city context, which may affect generalizability. External validation with data from other regions, using the sample code files supplemented, is needed to ensure the generalizability of the model. In addition, although the Random Forest model demonstrated robust performance across most antibiotics, imbalance-sensitive metrics indicated that predictions for antibiotics with extremely low resistance prevalence, such as Trimethoprim/Sulfamethoxazole, were less reliable, highlighting areas where future data collection or alternative modeling approaches could further improve predictive accuracy.

Low-prevalence antibiotics, such as Meropenem, were analyzed for completeness but should be interpreted as exploratory due to the very limited number of resistant cases. This distinction emphasizes the importance of cautious interpretation and highlights the need for future studies with larger, multicenter datasets to ensure reliable evaluation of these rare outcomes.

In addition, unlike studies employing explainable AI tools such as Shapley Additive exPlanations (SHAP) [16], our model has not yet incorporated feature attribution methods that could further enhance transparency in clinical decision making. Implementing such a rapid prediction system under real-world conditions in large hospital settings also presents several challenges. We identified four main obstacles that may influence the performance of the ML based decision systems. First, the integration with existing hospital information systems can be technically demanding, requiring substantial IT support. Second, the possible variability in microbiology laboratory practices, as well as differences in data quality and completeness, may impact model reliability. Third, successful implementation requires clinician training and user-friendly interfaces to build trust and ensure routine adoption of ML outputs in clinical workflows. Finally, financial and logistical constraints, including hardware requirements, software maintenance, and the need for dedicated IT support, may limit scalability in resource-constrained healthcare environments. Addressing these challenges is essential for translating predictive models from proof-of-concept studies into routine clinical practice. Despite the challenges, we believe that deploying the system in synchrony with current methodologies and gradually transitioning it into an alternative to the active system represents a more sustainable and effective approach for hospital settings.

In conclusion, to best of our knowledge, this is the first ML-based prediction study conducted in Turkey using centralized laboratory data. Our findings substantiate the feasibility and effectiveness of leveraging structured clinical data for predictive modeling of antibiotic resistance in *E. coli*. To support real-world adoption, our model could be integrated into hospital workflows through embedding within laboratory information systems and linkage to electronic health records, with future prospective studies needed to evaluate turnaround time, clinician training requirements, and the harmonization of data standards across institutions. The results demonstrates that ML algorithms, particularly RF, can achieve high classification performance with operational simplicity and cost-effectiveness, making them well-suited for deployment in emerging and resource-limited healthcare environments. Future research directions should focus on external validation with multicentric datasets, integration of interpretable ML frameworks to ensure transparent clinical decision-making, cooperative analyzing with WGS data, and incorporation into real-time hospital information systems to enable dynamic surveillance of antibiotic resistance surveillance and strengthen antimicrobial stewardship efforts.

## 4. Materials and Methods

This study was conducted with the approval of the Scientific Research Ethics Committee of Istanbul Health Sciences University Ümraniye Training and Research Hospital, dated 7 August 2025 and numbered 264/2025. The aim was to implement ML algorithms using the antibiotic susceptibility test results and associated metadata of *E. coli* isolates obtained from postoperative samples referred by general surgery clinics to the Istanbul Laboratories Region 2 Unit Laboratory. Bacterial identification was performed using the VITEK MS v3.2 system (bioMérieux, Marcy-l’Étoile, France), based on matrix-assisted laser desorption ionization time-of-flight mass spectrometry (MALDI-TOF MS).

### 4.1. Dataset Information

The data used in this study were collected from clinical specimens submitted by surgical departments of 11 hospitals affiliated with the Istanbul Laboratories Region 2 Unit Laboratory to its central microbiology laboratory over 5-year period (1 January 2020–31 December 2024). These hospitals include: Göztepe Training and Research Hospital, Beykoz State Hospital, Haydarpaşa Training and Research Hospital, Ümraniye Training and Research Hospital, Sancaktepe Training and Research Hospital, Şile State Hospital, Siyami Ersek Thoracic and Cardiovascular Surgery Training and Research Hospital, Sultan Abdulhamid Han Training and Research Hospital, Üsküdar State Hospital, and Zeynep Kamil Women and Children’s Diseases Training and Research Hospital. Information on the grouping of the data and the number of samples are given in Table 2. To avoid potential bias arising from repeated measurements, only one isolate per patient was included in the analysis. When multiple isolates were available from the same patient, the first isolate was selected, and subsequent isolates were excluded.

### 4.2. Data Cleaning and Preprocessing

During the data cleaning process, antibiotic records with fewer than 10 observations were excluded from the analysis. As a result, 475 patient records were removed, reducing the total number of patients from 1166 to 691.

Regarding missing data, no missing values were observed in patient-related variables such as gender, year of sample collection, clinical department, or sample type. The 13 antibiotics initially included in the dataset. Furthermore, due to pronounced class imbalances, only 12 antibiotics were retained for use in the antibiotic resistance estimation models.

Given that the dataset comprised entirely categorical variables, One-Hot Encoding was applied to transform these variables into a numerical format suitable for subsequent machine learning analyses. The antibiotics used in the study and the number of samples categorized according to the antibiotic resistance status of the patients are detailed in Table 3.

### 4.3. Machine Learning

In this study, Python version 3.13 was utilized for the analysis. The antibiotic resistance of *E. coli* was predicted using three machine learning algorithms: RF, CatBoost, and NB. Given that all variables in the dataset were categorical, these algorithms were selected based on their suitability for categorical data: Random Forest for its robustness with categorical inputs, CatBoost for being an optimized gradient boosting algorithm specifically designed to handle categorical features without the need for extensive preprocessing and Categorical Naive Bayes for its computational efficiency and simplicity, particularly in handling high-dimensional categorical data. Random Forest was selected for its robustness and interpretability, while CatBoost was included due to its optimization for categorical features with minimal preprocessing. Naive Bayes was used as a lightweight baseline to benchmark the performance of more complex algorithms [32,33].

To correct class imbalance, the Synthetic Minority Over-Sampling Technique (SMOTE) was applied exclusively to the training dataset to prevent data leakage into the test set. A fixed random state was used to ensure reproducibility of results (random_state = 42). Default SMOTE parameters were adopted unless otherwise specified, with the resampling strategy adjusted according to the minority class distribution of each antibiotic model. Additionally, SMOTE was applied separately for each antibiotic clas-sification task. Model performance was evaluated under three different scenarios: (i) by splitting the dataset into training and test sets in a 70:30 ratio, (ii) using 5-fold cross-validation, and (iii) using 10-fold cross-validation.

Classification performance metrics including accuracy, precision, recall, and F1-score were calculated for each scenario. Among these, extra calculations were performed for scenario (iii), which typically provides more reliable and generalizable predictions due to the larger number of validation folds. Therefore, area under the receiver operating characteristic curve (AUC) and feature importance analysis were additionally conducted based on the results of the 10-fold cross-validation to identify the most influential features contributing to the prediction of antibiotic resistance.

To assess the potential influence of resistance to one antibiotic on the resistance profile of another, a multi-class classification approach was not employed. Instead, each antibiotic was modeled separately as a binary classification problem, where the resistance status of a specific antibiotic served as the target variable. The predictor variables included patient demographic characteristics and resistance outcomes for the remaining antibiotics. Model performance was evaluated by computing the average of standard classification metrics (accuracy, precision, recall, F1-score, and AUC) across all target antibiotics, thereby providing a comprehensive measure of predictive effectiveness.

## Figures and Tables

**Table 1:** Overview of recent machine learning approaches for predicting antimicrobial resistance in *E. coli*.

No	Study (Year)	Country	Data Type	Target Organism	ML Algorithms Used	Performance Metrics (AUROC)	Best Model	Summary of Method	Compared to Literature
1	Moradigaravand et al. (2018) [3]	UK	Pan-genome (WGS)	*E. coli*	GBDT, RF, LR	Acc: 0.91 (avg)	GBDT	Resistance prediction using genome and population structure data	More flexible than rule-based systems
2	Moran et al. (2020) [25]	UK	Demographics, Culture, Antibiotics	*E. coli*, *Klebsiella pneumoniae*, *Pseudomonas aeruginosa*	XGBoost	AUROC: 0.70	XGBoost	Clinical data used for prediction	Rapid clinical utility potential
3	Rich et al. (2022) [26]	USA	EHR/DST	Urine (with *E. coli*)	DT, Boosted LR, RF	AUROC: 0.57–0.66	RF	Outpatient and urine-based prediction	Low–medium AUROC with wide dataset
4	Corbin et al. (2022) [27]	USA	EHR/DST	Various (with *E. coli*)	LASSO, Ridge LR, RF, GBDT	AUROC: 0.64–0.74	GBDT	Broad patient cohort	Big-data-based analysis
5	Weis et al. (2022) [14]	Switzerland	MALDI-TOF MS	*Staphylococcus aureus*, *E. coli*, *Klebsiella pneumoniae*	LightGBM, DNN, LR	AUROC: 0.74–0.80	LightGBM	Rapid test with spectral data	Near-clinical application
6	Tzelves et al. (2022) [28]	Greece	Demographics, Culture, DST, Gram-stain	Urine (with *E. coli*)	MLR	AUROC: 0.77–0.87	MLR	Prediction with gram-stained urine samples	Rapid clinical utility potential
7	Sakagianni et al. (2023) [13]	Greece	Non-genomic clinical data	Various (review)	RF, SVM, GBM, NN	AUROC > 0.85	RF/GBM	Narrative review	Showed ML success with non-genomic data
8	Tran Quoc et al. (2023) [11]	Vietnam	EMR + AST	ICU mixed bacteria (with *E. coli*)	RF, XGBoost, LightGBM	AUROC: 0.89–1.00	XGBoost	Clinical data used for prediction	Success in LMIC context
9	Yang et al. (2023) [29]	USA	Demographics, Culture, Antibiotics	*E. coli*	LR, XGBoost, TabNet	AUROC: 0.66–0.81	XGBoost	Clinical data used for prediction	Rapid clinical utility potential
10	Shields et al. (2024) [30]	USA	Demographics, Culture, Antibiotics	*E. coli*	LASSO	AUROC: 0.66–0.72	LASSO	Clinical data used for prediction	Rapid clinical utility potential
11	López-Cortés et al. (2025) [16]	Chile	MALDI-TOF (VITEK MS)	*E. coli*, *Staphylococcus aureus*, *Klebsiella pneumoniae*	CatBoost, RF, SVM	AUROC: 0.91	CatBoost	Spectral data + SHAP interpretability	Explainable AI approach
12	Our Study	Turkey	Demographics, Clinic, Antibiogram	*E. coli*	RF, CatBoost, NB	AUROC: 0.87	RF	Clinical data used for prediction	Rapid clinical utility potential

**Table 2:** Demographics of study participants (n = 691).

Characteristics		n	(%)
Sample Clinics	General Surgery	691	100.00
Gender	Male	329	47.61
	Female	362	52.39
Sample Collecting Year	2020	102	14.76
	2021	151	21.85
	2022	168	24.31
	2023	153	22.14
	2024	117	16.93
Sample Type	Aspirate	146	21.Ara
	Urine	180	26.Nis
	Wound	225	32.56
	Other ^1^	140	20.26

^1^ anaerobe, bile fluid, blood, catheter, peritoneal, sputum, pleural and tissue biopsy culture.

**Table 3:** List of antibiotics used in the study and sample numbers according to their resistance status.

Group	Antibiotics Name	Resistance Status		
		Susceptible (Class 1)	Intermediate (Class 2)	Resistant (Class 3)
		**(n)**	**(n)**	**(n)**
Aminoglycosides	Amikacin	617	38	36
Carbapenems	Ertapenem	676	0	15
	Meropenem	687	0	4
Cephalosporins	Cefoxitin	533	50	108
	Cefuroxime	36	221	434
	Ceftazidime	301	36	354
	Ceftriaxone	300	0	391
	Cefepime	342	47	302
Fluoroquinolones	Ciprofloxacin	267	38	386
Folate Synthesis Inhibitor	Trimethoprim/sulfamethoxazole	388	0	303
β-Lactams/β-Lactamase Inhibitors	Amoxicillin/clavulanic acid	280	9	411
	Ampicillin	144	0	547
	Piperacillin/tazobactam	528	20	143

## Data Availability

The patient-based antibiogram data are unavailable due to privacy or ethical restrictions.

## Supplementary Materials

The following supporting information can be downloaded at: https://www.mdpi.com/article/10.3390/antibiotics14100969/s1.

## Abbreviations

The following abbreviations are used in this manuscript:

AST	Antimicrobial susceptibility testing
AMR	Antimicrobial Resistance
BoostedLR	Boosted Logistic Regression
CatBoost	Categorical Boosting
DNN	Deep Neural Network
DT	Decision Tree
ESBL	Extended-spectrum beta-lactamases
GBDT	Gradient Boosted Decision Trees
GBM	Gradient Boosting Machine
KNN	K-Nearest Neighbors
LASSO	Least Absolute Shrinkage and Selection Operator
LightGBM	Light Gradient Boosting Machine
LR	Logistic Regression
ML	Machine Learning
MLR	Multiple Logistic Regression
NB	Naive Bayesian
RF	Random Forest
SHAP	Shapley Additive exPlanations
SMOTE	Synthetic Minority Over-sampling Technique
SSI	Surgical Site Infection
SVC	Support Vector Classifier
SVM	Support Vector Machine
TabNet	Tabular Neural Network
UTI	Urinary Tract Infections
WGS	Whole Genome Sequence
XGBoost	Extreme Gradient Boosting
