# Supplementary figures and images for: Machine Learning Model for Predicting Multidrug Resistance in Clinical Escherichia coli Isolates: A Retrospective General Surgery Study
Source: Antibiotics (Basel). 2025 Sep 26;14(10):969. doi: 10.3390/antibiotics14100969 (PMC12561689; doi:10.3390/antibiotics14100969)

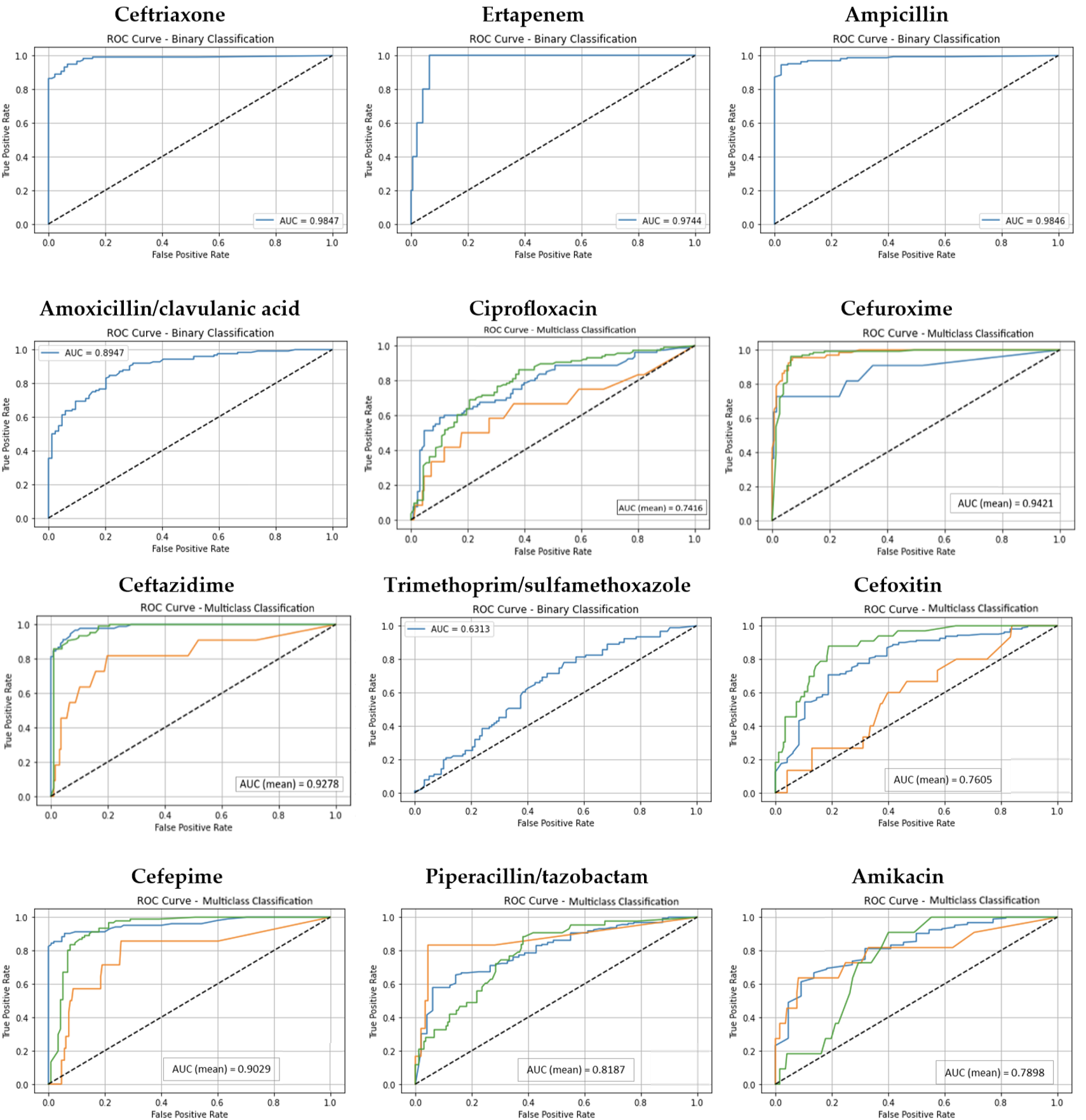

Supplement: Supplementary file 1 [file antibiotics-14-00969-s001.zip › Supplentary Figure S1.png]

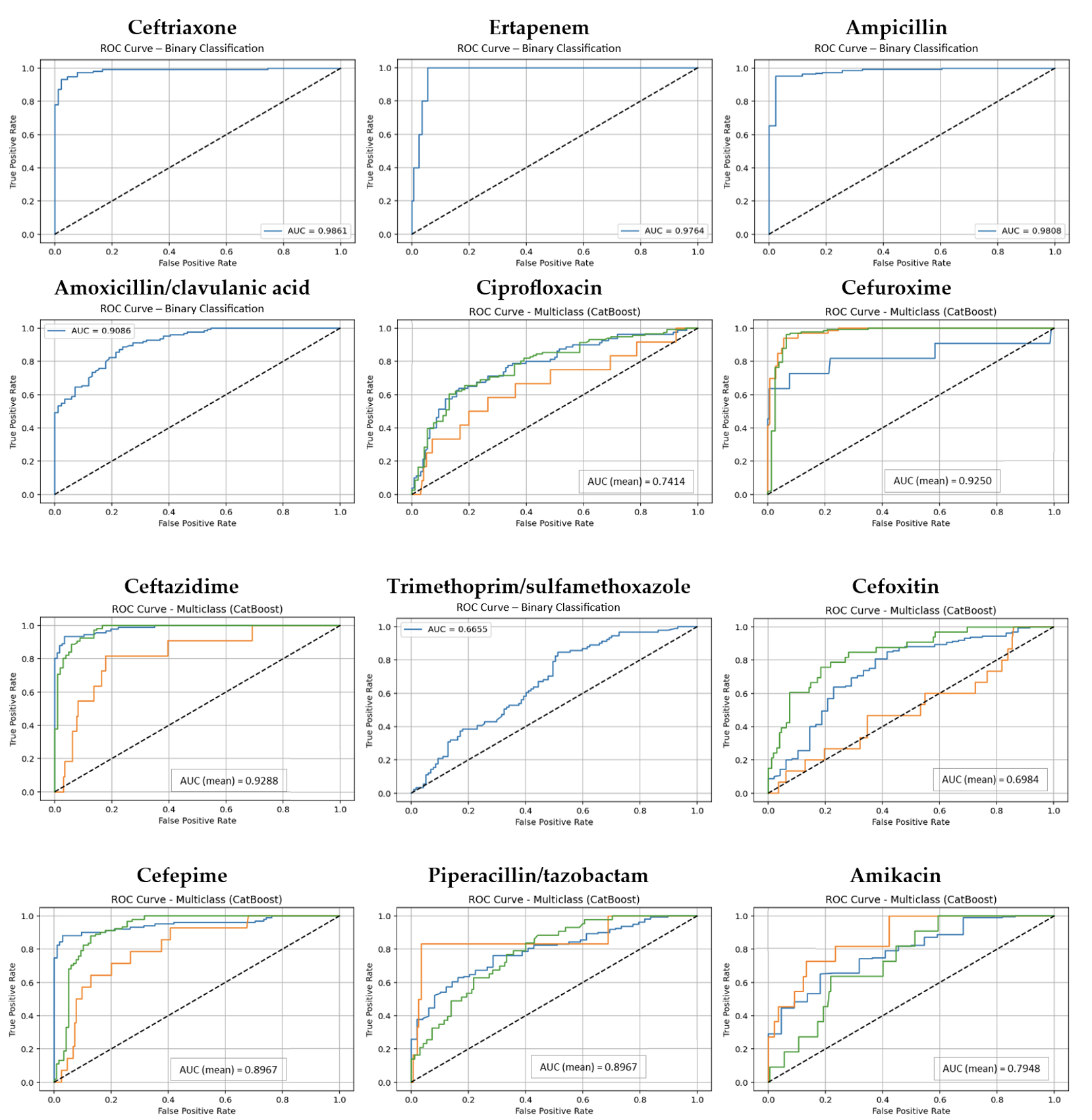

Supplement: Supplementary file 1 [file antibiotics-14-00969-s001.zip › Supplentary Figure S2.png]

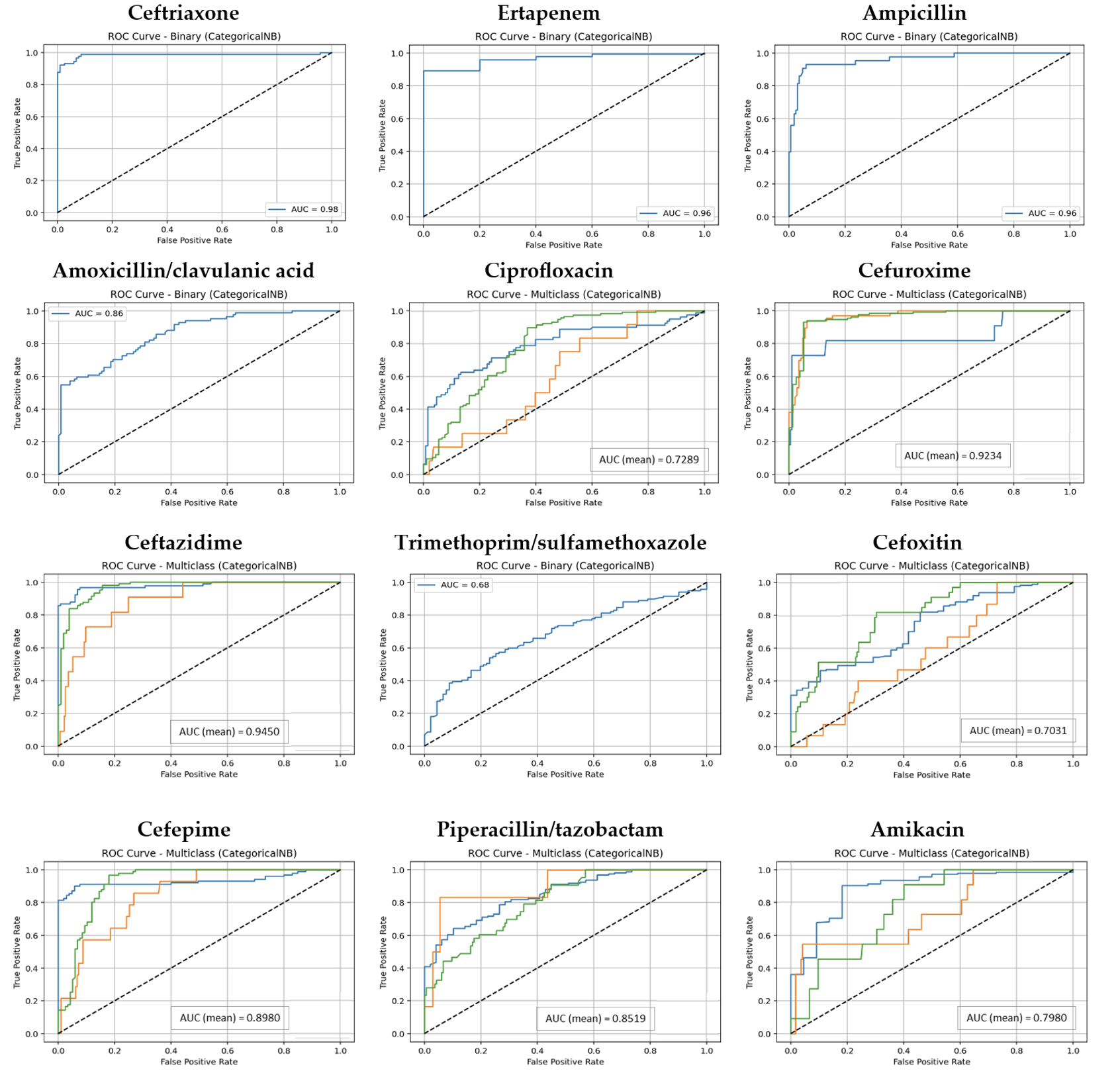

Supplement: Supplementary file 1 [file antibiotics-14-00969-s001.zip › Supplentary Figure S3.png]
